# New Evidence on the Role of D-Aspartate Metabolism in Regulating Brain and Endocrine System Physiology: From Preclinical Observations to Clinical Applications

**DOI:** 10.3390/ijms21228718

**Published:** 2020-11-18

**Authors:** Alessandro Usiello, Maria Maddalena Di Fiore, Arianna De Rosa, Sara Falvo, Francesco Errico, Alessandra Santillo, Tommaso Nuzzo, Gabriella Chieffi Baccari

**Affiliations:** 1 Dipartimento di Scienze e Tecnologie Ambientali, Biologiche e Farmaceutiche, Università della Campania «L. Vanvitelli», Via Vivaldi 43, 81100 Caserta, Italy; mariamaddalena.difiore@unicampania.it (M.M.D.F.); sara.falvo@unicampania.it (S.F.); alessandra.santillo@unicampania.it (A.S.); nuzzo@ceinge.unina.it (T.N.); 2 CEINGE Biotecnologie Avanzate, Via Gaetano Salvatore 486, 80145 Napoli, Italy; derosaar@ceinge.unina.it; 3 Department of Experimental Medicine, Sapienza University of Rome, 00185 Rome, Italy; 4 Dipartimento di Agraria, Università degli Studi di Napoli Federico II, Via Università, 100, 80055 Portici, Italy; erricof@ceinge.unina.it

**Keywords:** D-aspartate, D-aspartate oxidase, NMDA receptors, hormones, endocrine glands

## Abstract

The endogenous amino acids serine and aspartate occur at high concentrations in free D-form in mammalian organs, including the central nervous system and endocrine glands. D-serine (D-Ser) is largely localized in the forebrain structures throughout pre and postnatal life. Pharmacologically, D-Ser plays a functional role by acting as an endogenous coagonist at N-methyl-D-aspartate receptors (NMDARs). Less is known about the role of free D-aspartate (D-Asp) in mammals. Notably, D-Asp has a specific temporal pattern of occurrence. In fact, free D-Asp is abundant during prenatal life and decreases greatly after birth in concomitance with the postnatal onset of D-Asp oxidase expression, which is the only enzyme known to control endogenous levels of this molecule. Conversely, in the endocrine system, D-Asp concentrations enhance after birth during its functional development, thereby suggesting an involvement of the amino acid in the regulation of hormone biosynthesis. The substantial binding affinity for the NMDAR glutamate site has led us to investigate the in vivo implications of D-Asp on NMDAR-mediated responses. Herein we review the physiological function of free D-Asp and of its metabolizing enzyme in regulating the functions of the brain and of the neuroendocrine system based on recent genetic and pharmacological human and animal studies.

## 1. Introduction

During recent decades the development of increasingly more sensitive analytical methods has highlighted the presence of free D-aspartic acid (D-Asp) in the central nervous system (CNS) and in the endocrine system of the major vertebrate classes, including humans [1,2]. In these tissues, D-Asp results from: (1) racemization of L-aspartate [3]; (2) degradation of dietary protein; and (3) microbial synthesis in the intestine [4]. Nervous and endocrine tissues appear to contain the enzymatic systems required to modulate D-Asp homeostasis because they can synthesize and degrade this amino acid. Endogenous D-Asp racemase activity contributes to the biosynthesis of D-Asp from L-Asp, while D-aspartate oxidase (DDO), a peroxisomal flavoprotein that specifically metabolizes D-Asp into oxaloacetate, NH_3_, and H_2_O_2_ [5,6,7,8,9].

D-Asp has a different pattern of occurrence in mammalian tissues. Indeed, in the CNS, it is highly enriched during its prenatal development and decreases at birth [10,11]. Conversely, the concentration of this amino acid is low in the endocrine glands during gestational phases and progressively increases during postnatal life [12]. Pharmacologically, D-Asp can modulate glutamatergic NMDAR-mediated transmission and functions, and dysregulation of its metabolism occurs in the brain of schizophrenia patients and in an animal model of autism spectrum disorders [13,14,15].

Beyond the CNS, D-Asp function is known to regulate the synthesis and secretion of several hormones in endocrine and neuroendocrine tissues [2,6,16,17]. It induces the release of prolactin (PRL) by the anterior pituitary gland, modulates the production of oxytocin and vasopressin in the posterior pituitary gland, and suppresses the secretion of melatonin in the pineal gland. Furthermore, D-Asp regulates the synthesis and release of testosterone by promoting the release of gonadotropin-releasing hormone (GnRH) in the hypothalamus and luteinizing hormone (LH) in the pituitary. D-Asp can promote animal reproduction also by directly activating spermatogonia proliferation and improving sperm quality [18,19].

Herein we review current knowledge on the physiological function of D-Asp in the CNS and endocrine systems, particularly in terms of the molecular and physiological mechanisms underlying its activity. We also provide an overview of the therapeutic potential of D-Asp in human health.

## 2. Free D-Aspartate Distribution in the Mammalian Central Nervous System

D-amino acids occur in microorganisms, plants, and animals [1]. Free D-Asp in mammals was first discovered in rats and humans at the end of the 1980s [20]. This atypical amino acid has been found in the CNS of rats [20,21], mice [22,23,24], and humans [10,13,25]. Although amino acids are predominantly present in mammalian tissues in the L-form, D-Asp content in the human embryonic prefrontal cortex (PFC) exceeds even the amount of its enantiomer, L-Asp (mean values: D-Asp = 0.036 μmol/g, L-Asp = 0.21 μmol/g), while the levels of this D-amino acid are drastically reduced at adulthood (0.008 μmol/g) [9,10,25,26]. In line with this finding, immunohistochemical studies in the rat embryonic brain revealed that D-Asp appears within the hindbrain at embryonic day (E) 12, and then in the mid and forebrain at E20. In terms of cellular localization, D-Asp undergoes peculiar developmental-related changes: it was first found to be restricted to the cytoplasm of neuroblasts after which it occurred in axonal terminals [11]. These neuroanatomical observations support the hypothesis that D-Asp is involved in neuronal differentiation [27]. Studies of rat brain showed that D-Asp is localized at birth in the cerebral cortex, hippocampus, and cerebellum [28]. At postnatal day (P) 7, the concentration of D-Asp progressively decreases and almost disappears in one-month-old rats [28,29]. In line with immunohistochemical studies, high-performance liquid chromatography (HPLC) analyses showed that the D-Asp/total Asp ratio dramatically decreases after birth in human, rat, and mouse brain regions, and persists at very low levels during adulthood [24,28,30]. Interestingly, the extraordinary gestational abundance of the D-Asp/total Asp ratio significantly differs between the human and rodent nervous systems. Indeed, a recent study in mice showed that D-Asp levels never reach as high as 12% of total embryonic Asp [22], whereas in the human PFC homogenates at gestational week 14, its relative abundance is around 65% [10].

The cellular biosynthesis of D-Asp in mammals was first identified in pheochromocytoma (PC12) cells [31]. Subsequently, D-Asp synthesis was identified in rat pituitary tumor GH_3_ cells [32] and in human cervical adenocarcinoma HeLa cells [33], whereas D-Asp generation in mammalian tissue remains a matter of debate. Kim et al. suggested a pyridoxal 5 phosphate (PLP)-dependent glutamate-oxaloacetate transaminase 1-like 1 (Got1l1) as the enzyme that converts L-Asp to D-Asp in the rat brain [34]. However, the finding of comparable D-Asp levels in the hippocampus of wild-type and Got1l1 knockout mice suggested that an, as yet unknown, D-aspartate-synthesizing enzyme is responsible for D-Asp biosynthesis in the mammalian brain [35]. An alternative biochemical pathway for D-Asp synthesis has also been proposed in mammals and involves serine racemase (SRR), a well-known enzyme related to D-Serine (D-Ser) metabolism [36,37]. Consistently, D-Asp levels were found to be reduced in the forebrain but not in the cerebellum of D-*Srr* knockout mice, which suggests that this enzyme is involved in D-Asp synthesis in specific brain subregions [36,37]. On the other hand, it has long been known that DDO is the degradative enzyme responsible for D-Asp catabolism [23,25]. In fact, DDO is a peroxisomal flavoprotein that catabolizes the oxidative deamination of D-Asp to generate α-oxaloacetate, hydrogen peroxide, and ammonia (for more recent insights on DDO biochemical properties and structure-functional relationship in different species, see the reviews [9,38,39,40]). The intracellular localization of DDO in organelles like peroxisomes enables the cell to safely contain the hydrogen peroxide produced by the degradative reaction [41]. Remarkably, this enzyme is expressed in the CNS and is active during postnatal life mainly within the neuronal population [41,42,43]. Recent molecular studies in the mammalian brain suggested that the postnatal expression of DDO is regulated at transcriptional level because *Ddo* mRNA expression increases greatly throughout the mouse brain from the early embryonic life to the adult stage [24,44]. The latter finding led to the identification of the epigenetic events underlying the temporal regulation of *Ddo* gene expression. In this context, it has recently been demonstrated that the dynamic changes in cerebral *Ddo* transcription are closely associated with progressive active demethylation of its promoter region [24,25]. Additionally, this finding led to clarify the effect exerted by DNA methylation on the transcription of *Ddo* and of other genes involved in D-Ser metabolism, namely, *D-amino acid oxidase* (*Daao*), *Srr*, and *G72* [45], in both mouse and human brain [30,46,47,48]. In particular, the analysis of specific combinations of methylated CpG islands, defined “epialleles”, indicated that neurons, oligodendrocytes, astrocytes, and microglial have a cell type-specific methylation pattern at the *Ddo* promoter [30].

## 3. Pharmacological Features of D-Aspartate

In the 1980s, neuropharmacological studies showed that D-Asp binds to the L-glutamate (L-Glu) site of ionotropic NMDA receptors (NMDARs) [49,50]. More recently, in line with radioligand binding studies, electrophysiological experiments demonstrated that local applications of D-Asp on mouse brain slices triggered inward currents that were antagonized by the competitive and noncompetitive NMDAR blockers, D-2-Amino-5-phosphonovaleric acid (D-AP5) and (+)-5-methyl-10,11-dihydro-5H-dibenzo[a,d]cyclohepten-5,10-imine maleate (MK801), respectively [51,52]. However, residual D-Asp-dependent inward currents persisted even after the application of high doses of these NMDA antagonists [51,53], which suggests that this amino acid is able to bind and activate other receptor complexes [53]. Subsequent pharmacological studies showed that D-Asp also inhibits kainate-induced α-amino-3-hydroxy-5-methyl-4-isoxazolepropionic acid-type glutamate receptor (AMPAR) currents in rat hippocampal neurons and stimulates metabotropic glutamate receptor 5 (mGluR5) in mouse and rat brain slices [54,55,56].

Similar to hippocampus, striatum, and spinal cord [57], application of D-Asp triggered inward currents in dopamine neurons of substantia nigra pars compacta by activating NMDAR and, to a lesser extent, of AMPA and mGlu1/5 receptors [52]. Studies of primary neuronal cultures and synaptosomal preparations have demonstrated that D-Asp is stored in secretory organelles and released from axon terminals through vesicular exocytotic processes mediated by Ca^2+^ [28,58] or probably by spontaneous release or L-Glu transporter exchange [59,60]. Notably, very recently microdialysis experiments showed that D-Asp efficiently crosses the blood brain barrier, and most importantly, it is detectable at nanomolar concentrations in the extracellular space within the prefrontal cortex of freely-moving mice [56,61]. The latter experiment also demonstrated that free D-Asp is released by neurons through a Ca^2+^-dependent mechanism [56]. As reported in mammals, extracellular D-Asp has also been found in the brain of domestic chicks, where it decreases in an age-dependent manner and is transiently induced following high K^+^ stimulation [3]. Lastly, it has been suggested that the intracellular uptake of D-Asp may depend on L-Glu/L-Asp transporter systems that recognize L-Glu and both Asp enantiomers [62]. Furthermore, recent in vitro studies revealed that D- and L-Asp are recognized and transported by the glutamate transporter homolog Glt_Tk_ in the same way and with comparable affinity [63].

## 4. Animal Models with Altered D-Aspartate Metabolism

At the beginning of the 2000s, two laboratories generated *Ddo* knockout (ko) mice (*Ddo*^−/−^*)* to elucidate the biological function of *Ddo* and its substrate, D-Asp, in the endocrine and nervous systems [51,64,65]. HPLC analysis of the hippocampus, striatum, cortex, cerebellum, olfactory bulbs, and peripheral organs of *Ddo* ko mice revealed selective increases of D-Asp content (~10- to 20-fold) versus wild-type (*Ddo*^+/+^) littermates while L-Asp and L-Glu levels remained unaltered [24,44,53,64].

Moreover, because D-Asp is converted into NMDA by D-aspartate methyltransferase using S-adenosylmethionine as a methyl donor [66], a significant increase of endogenous NMDA was also observed in *Ddo*^−/−^ brain homogenates [44,53]. In line with previous studies carried out on cerebral homogenates [24,44,51,53,67,68], we found that extracellular D-Asp levels were higher in the PFC of freely moving *Ddo*^−/−^ mice than in wild-type mice, which suggests that impaired catabolism of this amino acid dramatically affects its extracellular concentration [24]. In addition to *Ddo* gene removal, acute and chronic treatment with D-Asp in mice and rats were used to raise the cerebral levels of this amino acid. In detail, treatment with oral D-Asp treatment in tap water for one month caused an extracellular increase of D-Asp in mouse brain [51,53,56]. Similarly, acute intraperitoneal (i.p.) administration of 500 mg/kg D-Asp caused a rapid transient increase in D-Asp content in C57BL6J mice as early as 20 min post-injection [56]. More recently, Kitamura et al. demonstrated that, in the rat, gastric gavage of D-Asp reaches the hippocampus via blood circulation as early as 15 min post-administration [69]. The latter results confirmed that exogenous D-Asp crosses the blood-brain barrier and reaches the brain parenchyma, as previously reported only for two D-amino acids: D-Ser and D-proline [70,71]. Interestingly, both chronic oral administration and acute i.p. injection also evoke cortical L-Glu efflux in freely moving animals, probably by stimulating presynaptic glutamate receptors (NMDAR, AMPAR, and mGluR5) [56]. The afore body of data suggests that D-Asp is a central regulator molecule within glutamatergic system, that acts by activating this neurotransmission, on one hand directly by postsynaptic NMDAR activation while, on the other, evoking L-Glu efflux from cortical neurons.

In agreement with the effect exerted by D-Asp on glutamatergic neurotransmission, electrophysiological studies performed in the CA1 area of the hippocampus of adult *Ddo*^−/−^ and D-Asp-treated mice showed that increased D-Asp levels enhance NMDAR-dependent early-phase and late-phase long-term potentiation [51,53,72]. Moreover, in line with the role of D-Asp as an NMDAR agonist, the frequency of NMDAR-mediated miniature excitatory postsynaptic currents in pyramidal neurons of the medial PFC layer II/III is enhanced in mice chronically treated with this amino acid [72]. In addition, D-Asp supplementation triggered pronounced metabolic activity in cortical and hippocampal areas as measured by functional magnetic resonance imaging [72]. In line with this observation, 10–20 min after stomach gavage in awake rats, D-Asp increased slow-frequency synchronization in the hippocampus, somatosensory cortex, striatum, and nucleus accumbens. The latter findings suggest that D-Asp controls hippocampal and cortical neural network activity [69].

Abnormal levels of D-Asp affect synaptic morphology in mice brain [72]. In fact, increased D-Asp levels augmented dendritic length and spine density in the PFC and hippocampus of chronically D-Asp-treated and *Ddo*^−/−^ mice [72]. Consistent with mouse findings, in vitro studies showed that exposure of rat hippocampal slices to D-Asp significantly increases the density of the middle size spines of hippocampal neurons via an actin-sensitive mechanism [69]. In line with enhanced NMDAR-dependent transmission and facilitated induction of the late phase of synaptic plasticity, spatial cognitive function was improved in *Ddo*^−/−^ and D-Asp-treated mice as evaluated by the hidden platform version of the Morris water maze (MWM) test and contextual fear conditioning [51,53]. Similar behavioral performances have been described in D-Asp-treated rats [73].

Overactivation of NMDARs causes glutamate excitotoxicity and cell death [74,75]. Hence, consistent with its pharmacological features, abnormally high D-Asp levels can detrimentally affect the brain through NMDAR overstimulation. Indeed, 13/14-month-old *Ddo*^−/−^ mice had severe deficits of synaptic plasticity, spatial learning, and memory [53]. Additionally, high D-Asp levels in primary cortical neurons and *Ddo*^−/−^ mutants induces severe neuroinflammation processes and cell death [24,76], which indicates that DDO activity prevents detrimental cerebral NMDAR overstimulation and ultimately neuron death.

Given that NMDARs regulate neuron development, maturation, and migration [77,78,79,80], we argue that D-Asp is a candidate signaling molecule involved in neurodevelopmental processes. Notably, a recent study revealed a severe reduction of *Ddo* gene expression accompanied by an increase of D-Asp levels in the brain of BTBR mice, which is a widely accredited animal model of idiopathic autism [14]. Furthermore, in an attempt to understand the biological meaning of the elevated levels of D-Asp during brain development, a *Ddo* knock-in mouse model with complete depletion of this D-amino acid has been generated [22]. Interestingly, *Ddo* knock-in mice are viable, fertile, and have normal gross brain morphology in adult stage; however, they have paradoxically enhanced memory abilities as evaluated by the object recognition and MWM tests associated to an altered number of cortical parvalbumin-positive interneurons [22].

## 5. D-Aspartate Metabolism Alteration in Neurological and Psychiatric Disorders

Based on the impact of dysfunctional NMDAR and mGluR5 transmission in neurological and psychiatric disorders [81,82,83,84,85,86], various groups investigated the involvement of altered D-Asp metabolism in patients affected by some of these conditions and in respective animal models. Altered glutamatergic transmission has long been implicated in Parkinson’s disease (PD) and in motor complications caused by L-3,4-dihydroxyphenylalanine (L-DOPA) therapy [87,88]. Recently, D-Asp content was found to be dramatically upregulated in the putamen of a primate model of PD [89]. Moreover, D-Asp levels were found to be similar in the serum and cerebrospinal fluid of diverse diagnosis groups, while D-Ser concentrations were significantly lower in L-DOPA-free PD patients [89].

D-Asp and, to a greater extent, D-Ser content have also been investigated in another neurodegenerative disorder, namely, Alzheimer’s disease (AD) because they may reflect dysfunctional activation of neuronal glutamatergic NMDAR. In this context, it is noteworthy that D-Ser and D-Asp metabolism evaluated in the serum and cerebrospinal fluid of a large cohort of drug-free subjects encompassing the whole AD clinical spectrum did not differ from that of age-matched controls [90]. Similarly, postmortem analysis showed comparable D-Asp and D-Ser levels in superior frontal gyrus samples in AD patients and nondemented controls [76,90].

Finally, D-Asp metabolism has been also investigated in patients affected by schizophrenia. In particular, two postmortem studies showed a significant D-Asp reduction in the PFC of these patients associated with increased *DDO* gene expression [15] or enzymatic DDO activity [13].

## 6. Free D-Aspartate Distribution in Neuroendocrine and Endocrine Systems

Mammalian endocrine glands in adulthood contain not negligible levels of free D-Asp and possess the enzymes for the homeostasis of this amino acid [2,6,91]. In particular, DDO occurs in the pineal and pituitary glands in both corticotropic (adrenocorticotropic hormone (ACTH)-producing) and melanotropic (proopiomelanocortin (POMC)-producing) cells [29,92]. Moreover, DDO has been found in the adrenal gland [29], thyroid gland [93], and testis [94,95]. In line with the degradative activity of DDO, the levels of D-Asp were found to be higher in several endocrine tissues (hypothalamus, pituitary gland, pineal gland, pancreas, adrenal gland, and ovary) of mutant mice with targeted *Ddo* deletion than in control mice [64,96]. Conversely, D-Asp levels were unaltered in the hypothalamus and pituitary of mutant mice with targeted deletion of *Daao*, while the D-Asp concentration in the pineal gland was surprisingly higher in the mutants than in the controls [97,98,99].

Unlike in the mammalian CNS, endogenous D-Asp levels in some endocrine glands, namely, the pituitary gland [12,100], pineal gland [101], adrenal gland [102], and testis [2] significantly increase during postnatal development and then gradually become stable when the organ is completely differentiated. This observation suggests that D-Asp might play a role in the maturation and function of these organs [12]. In line with this view, in adult mammals, the intraperitoneally or orally administered D-Asp accumulates in the endocrine glands and acts as an excitatory molecule by inducing the synthesis and secretion of different hormones [12,17].

In the rat hypothalamus, D-Asp is mostly concentrated within the magnocellular neurons of the supraoptic and paraventricular nuclei [29,103]. Moreover, increased D-Asp levels following its administration triggered the synthesis of oxytocin [27,104] and inhibited the release of dopamine [105] in this brain area. Furthermore, D-Asp supplementation induced the release of the luteinizing hormone release hormone (LHRH), α-melanocyte-stimulating hormone (α-MSH), and gamma-amino butyric acid (GABA) and also increased the activity of nitric-oxide synthase (NOS) [106]. The effect of D-Asp supplementation on hypothalamic GABA release is in agreement with the influence that another D-amino acid, D-Ser, has on GABA transmission in the brain. Indeed, a recent preprint study has shown that D-Ser could greatly influence the excitation/inhibition balance within neuronal networks and that the absence of this D-amino acid disrupts this balance, at least through reduced inhibitory GABA connections [66]. Taken together, these observations suggest that D-Asp and D-Ser could both play a role in regulating neuroendocrine GABA-mediated transmission.

Among endocrine tissues, the pineal gland is the one that contains the highest amounts of D-Asp [98,107,108,109,110,111]. In rats, pineal D-Asp levels undergo remarkable changes in relation to the circadian biorhythm, being higher at night than in the day [101]. Moreover, immunohistochemical and biochemical analyses revealed a high D-Asp concentration in melatonin-secreting pinealocytes [29,112]. Rat pinealocytes pretreated with D-Asp released D-amino acid in response to norepinephrine stimulation, while norepinephrine-dependent melatonin secretion was suppressed through the cAMP inhibitory cascade [109,113]. In contrast to these results, which suggest that D-Asp acts as a negative regulator of melatonin synthesis, Han and coworkers [108] reported a positive correlation between D-Asp concentration and melatonin amount in the pineal gland of diverse rodent strains.

In the rat adrenal gland, D-Asp was found to be selectively concentrated in epinephrine-producing chromaffin cells [29,98,114]. In adrenal sections, D-Asp is released through activation of the cholinergic innervation [28]. Interestingly, rat pheochromocytoma PC12 and MPT1 cells were found to contain D-Asp in dopamine-containing secretory granules that were secreted through Ca^2+^-dependent exocytosis [115,116]. Finally, in *Ddo*^−/−^ mice, cells of the adrenal gland showed immunoreactivity to D-Asp, which was not detected in wild mice (Weil et al. 2006). As reported in the brain and spinal cord [25], there are compelling evidences that D-Asp-related effects in endocrine glands are mediated by L-Glu receptors [117,118].

## 7. D-Asp Administration on the Hypothalamus-Pituitary-Testis Axis

Experimental studies performed in various animal models have shown that D-Asp acts at all levels of the hypothalamic-pituitary-testis axis, which suggests that this D-amino acid plays a role also in vertebrate reproductive processes [16,17,18,19,91].

D-Asp has been found in substantial amounts in the mammalian pituitary gland [92,100,110,119,120]. In rat pituitary, the highest amount of D-Asp occurred in the adenohypophysis, particularly in PRL-secreting cells [114]. Remarkably, pituitary D-Asp levels were boosted by estrogen implant, which in turn increased the number of PRL-producing cells [121]. However, D-Asp supplementation in rats and sheep also resulted in a significant increase in LH, PRL, and in growth hormone levels [93,103,105,122]. Accordingly, Topo and coworkers [93] reported that supplementation with D-Asp in humans leads to an increase in serum LH levels. In addition, high levels of D-Asp in the pituitary intermediate lobe of *Ddo*^−/−^ mutant mice have been correlated with a reduction in the levels of α-MSH, which leads to alterations of the POMC/α-MSH as well as melanocortin-mediated behaviors, which suggests that D-Asp plays a physiologic role in sexual behavior [64].

**Figure 1:**
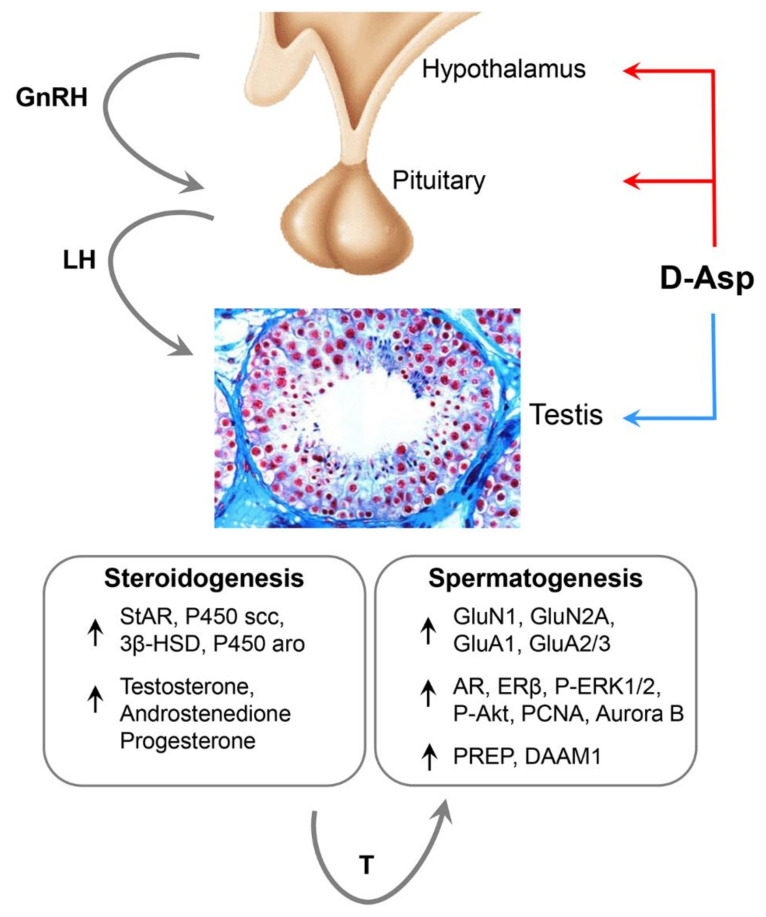
Schematic representation of D-Asp effect on hypothalamic-pituitary-testis axis. D-Asp regulates spermatogenesis at two different levels. It acts (1) on the hypothalamic-pituitary-testis axis (red arrow) by eliciting the release of gonadotropin-releasing hormone (GnRH), luteinizing hormone (LH), and testosterone (T); (2) directly on the testis (blue arrow) by inducing T release. In the Leydig cells, D-Asp modulates steroidogenesis by eliciting the expression of the steroidogenic acute regulatory protein (StAR), cytochrome P450 cholesterol side-chain cleavage (P450scc), 3β-hydroxysteroid dehydrogenase (3β-HSD), and cytochrome P450 aromatase (P450 aro) steroidogenic enzymes. In testis, D-Asp activates NMDA (GluN1 and GluN2A subunits) and AMPA (GluA1 and GluA2/3 subunits) receptors. D-Asp enhances androgen receptor (AR) and estrogen receptor β (ERβ) expressions and induces spermatogonial proliferation by increasing PCNA and Aurora B expressions via ERK and Akt pathways. Finally, D-Asp increases the expression of prolyl endopeptidase (PREP) and disheveled-associated activator of morphogenesis 1 (DAAM1), two proteins involved in the cytoskeleton remodeling.

The hypothalamus gland of various mammalian species possesses considerable concentrations of D-Asp and the ability to accumulate it following its administration [106]. In rat and mouse testis, D-Asp levels are low at birth and then gradually increase starting at 7 weeks of age and remain relatively constant in adults [2,12,98,108]. Immunohistochemical studies have shown that D-Asp is localized in germ cells as well as in Leydig cells [102,106]. Interestingly, oral or i.p. administration of D-Asp in both male rats [103] and sheep [122] induced an increase in serum LH levels. This finding led to the hypothesis that D-Asp stimulates the release of GnRH from the rat hypothalamus, which, in turn, induces the release of LH from the pituitary gland ultimately resulting in increased testosterone biosynthesis (Figure 1). An increase in serum androstenedione and progesterone levels has also been detected [93,103,117,123,124]. Similarly, treatment with D-Asp in mice induced a significant increase in serum and testis LH, testosterone, and epitestosterone [18,19].

The increase in sex steroid hormone levels results from the effect of this D-amino acid on the transcription of the acute steroidogenic regulatory protein (StAR), which is a primary regulatory protein for the biosynthesis of testosterone in the testis as well as of steroidogenic enzymes (Figure 1) [117,124]. In accordance with these findings, in vivo and in vitro studies showed that D-Asp modulates the levels of the biologically active sex hormones also in the brain by upregulating the activities of steroidogenic enzymes [125,126,127]. Even in the epididymis, an organ in which an initial phase of sperm maturation occurs, D-Asp modulated the levels of androgens and estrogens by acting on the expression of the genes *5α-reductase* and P450-aromatase family member, *Cyp19a1*, respectively [128]. On the other hand, the increased expression of androgen and estrogen receptors, observed in testis or epididymis of D-Asp-treated rats, further supports that D-amino acid plays a regulatory role in spermatogenesis.

The effects of D-Asp on the hypothalamic-pituitary-testis axis found in animal models have been confirmed by the results of in vitro experiments. Indeed, D-Asp induced LH release by isolated adenohypophysis or adenohypophysis co-incubated with hypothalamus by cyclic guanosine monophosphate (cGMP) pathway [93,103,123]. D-Asp alone or in the presence of human chorionic gonadotropin upregulated the production of androstenedione and testosterone in both immature [124] and mature Leydig cells [129] by inducing the expressions of StAR, P450scc, and 3β-HSD. A recent study on Leydig murine cells showed that treatment with D-Asp alone did not induce any significant changes in testosterone release or in LH receptor protein expression, whereas the addition of human chorionic gonadotropin significantly increased both StAR protein gene expression and testosterone levels [130].

Functionally, it is plausible that D-Asp affects the hypothalamic-pituitary-testis axis through L-glu receptors, including the ionotropic receptors NMDAR and AMPAR. NMDARs were found to be expressed in hypothalamic GnRH-secreting neurons of rats as well as in pituitary hormone-secreting cells [105,131]. Furthermore, the presence of functionally active glutamate receptors has been demonstrated in the testes of both rats and humans [132].

In rat testis, D-Asp induces testosterone synthesis and upregulates androgen receptor expression throughout NMDAR activation (Figure 1) [117,133]. In addition, short-term culture of spermatogonia GC1 cells showed that D-Asp activates the ERK/Aurora B proliferative pathway through AMPAR (Figure 1) [134,135]. Conversely, Tomita and coworkers showed that in long-term culture (14–21 days) of the isolated mouse testis, exogenous D-Asp suppresses germ cell differentiation by reducing the number of mitotic and meiotic cells [136]. The discrepancy between the results of these studies could be due to the different experimental approaches.

Further confirmation that D-Asp plays an active role in spermatogenesis is the increase in the expression of prolyl endopeptidase (PREP) [137] and disheveled-associated activator of morphogenesis 1 (DAAM1) [138] in the testis of D-Asp-treated rats (Figure 1). These two proteins are involved in cytoskeleton remodeling, which is an integral aspect of spermatogenesis and is therefore essential for male fertility. In particular, PREP is a binding partner of tubulin in the cytoplasm of Sertoli cells, Leydig cells, and germ cells, and in the nucleus of spermatogonia and spermatocytes; DAAM1 is a protein of the formin family involved in the nucleation of unbranched actin filaments. Interestingly, treatment with D-Asp induced not only an increase in DAAM1 protein levels in the rat testis but also the migration of this formin from the cytosol to the nucleus in germ cells, suggesting a role for this amino acid as a regulator of both cytosolic and nuclear actin.

Finally, experiments on isolated mouse spermatozoa showed that D-Asp improved total and progressive sperm motility [18,19]. Furthermore, treatment with a commercial mixture of Coenzyme Q10, zinc, and D-Asp (CZA) exerts a protective effect on bovine spermatozoa, since it counteracts the loss of motility by acting on the mitochondrial membrane potential (MMP) and DNA fragmentation [17,139].

## 8. D-Asp Supplementation in Humans

It has been reported that the concentration of D-Asp was lower in the seminal plasma and spermatozoa of oligoasthenoteratospermic and azoospermic patients than in normospermic donors [140]. Treatment of subfertile patients with commercial products containing D-Asp (Dadavit^®^ or Genadis^®^: sodium D-Asp supplemented with folic acid, vitamins B6 and B12) improved the number and motility of spermatozoa [141]. After D-Asp supplementation, the concentration of spermatozoa in the oligoastenozoospermic patients was increased by about 2-fold and the motility of the spermatozoa by about 1.5-fold; in asthenozoospermic patients the sperm concentration was approximately 1.6-fold higher and the rapid progressive sperm motility was increased by about 1.9-fold [141].

An attempt to use D-Asp in assisted reproduction techniques has recently been made. The addition of CZA to the culture medium containing spermatozoa of sub-fertile patients prevented a reduction in sperm kinetics, particularly in oligospermic samples. Furthermore, CZA treatment protected spermatozoa from an increase in DNA fragmentation and lipid peroxidation [17,142]. However, the effect of the various components of the mixture is synergistic since none of the components used individually at various concentrations improved sperm performance versus controls.

Few studies have investigated the role of D-Asp in female fertility. D’Aniello and coworkers identified a negative relationship between the content of D-Asp in pre-ovulatory follicular ovarian fluid and the patient’s age [2]. D-Asp concentration was higher in younger women (19.11 ± 1.91 nmol/mL) than in older women (10.86 ± 1.22 nmol/mL). Lastly, the follicular concentration of D-Asp was found to be positively correlated to the percentage of good quality oocytes and to fertilization rate.

Despite the therapeutic potential of D-Asp found in preclinical models, the use of this amino acid in human fertility is still a matter of debate and very few clinical investigations have been performed. Topo et al. [93] showed that D-Asp supplementation (Dadavit^®^: 3,12 g/day D-Asp supplemented with folic acid and vitamins B6 and B12) in humans for 12 days increased the levels of LH and testosterone, respectively, to 33% and 42%. Three days after Dadavit^®^ suspension, while LH levels returned almost to baseline, testosterone levels remained significantly higher. A possible explanation of this finding is that ingested D-Asp remained in the testis where it continued to stimulate testosterone production.

Given the positive relationship among testosterone, GH levels, and muscle hypertrophy [143], studies by Willoughby and Leutholtz investigated the influence of D-Asp in modulating hypothalamus-pituitary-testis axis hormones and muscle strength in trained men [144]. The authors demonstrated that D-Asp (3 g/day) supplementation for 28 days in resistance-trained men aged 23–28 years with serum testosterone levels ~ 7.96 ng/mL is ineffective in up-regulating the activity of the hypothalamus-pituitary-gonadal axis and has no anabolic or ergogenic effects in skeletal muscle [144].

The discrepancy between Willoughby and Leutholtz [144] and Topo [93] results may reflect differences in age, state of training, and basal testosterone levels, which were higher in trained men. Specifically, basal testosterone levels in resistance-trained men ranged from around 5.8 to 8.6 ng/mL and in untrained men from around 4.9 to 6.6 ng/mL. In addition, Melville and coworkers [145] evaluated the effects of two doses of D-Asp (3 g and 6 g) administered for 2 weeks on basal testosterone levels. In line with Willoughby and Leutholtz [144], they confirmed that 3 g/day of D-Asp did not affect testosterone levels and that 6 g/day significantly reduced testosterone levels, without any concomitant change in estradiol. In another work, Melville and coworkers [146] showed that D-Asp 6 g/day in resistance-trained men over a 3-month training period did not change basal testosterone levels and caused a marked reduction (about 95%) in estradiol levels.

In addition to sex hormone-related effects, recent preclinical and clinical studies have highlighted the beneficial effect of D-Asp supplementation in mouse models and in patients with multiple sclerosis [147,148]. In particular, a recent clinical trial revealed that four-week oral D-Asp supplementation in multiple sclerosis patients enhances transcranial magnetic stimulation-induced LTP and intracortical facilitation, which suggests an improvement in synaptic plasticity reserve and trans-synaptic glutamatergic transmission [148].

## 9. Conclusions

Negligible levels of D-Asp in the mammalian CNS led to the incorrect interpretation that D-Asp is devoid of any physiological role. However, preclinical research over the last two decades has shown that D-Asp affects neural and neuroendocrine signaling with implications for human health.

Despite many advances since Hashimoto’s groundbreaking observation [10], there are still many opened questions regarding the functions of D-Asp in the CNS and in the peripheral nervous system. Concerning the function of D-Asp in the endocrine system, the bulk of information from preclinical studies strongly suggests that this D-amino acid plays a key role in mammalian reproductive processes. However, there is still a need to decipher the specific contribution of this atypical amino acid in healthy and in the diseased nervous and endocrine systems.

## References

[1] Genchi G. (2017). An overview on d-amino acids. Amino Acids.

[2] D’Aniello G., Grieco N., Di Filippo M., Cappiello F., Topo E., D’Aniello E., Ronsini S. (2007). Reproductive implication of D-aspartic acid in human pre-ovulatory follicular fluid. Hum. Reprod..

[3] Fleck M.W., Barrionuevo G., Palmer A.M. (2001). Synaptosomal and vesicular accumulation of l-glutamate, l-aspartate and d-aspartate. Neurochem. Int..

[4] Bastings J.J., Van Eijk H.M., Damink S.O., Rensen S.S. (2019). d-amino Acids in Health and Disease: A Focus on Cancer. Nutrients.

[5] D’Aniello A., Vetere A., Petrucelli L. (1993). Further study on the specificity of d-amino acid oxidase and of d-aspartate oxidase and time course for complete oxidation of d-amino acids. Comp. Biochem. Physiol. Part B: Comp. Biochem..

[6] Di Fiore M.M., Santillo A., Chieffi Baccari G. (2014). Current knowledge of d-aspartate in glandular tissues. Amino Acids.

[7] Katane M., Homma H. (2010). D-Aspartate Oxidase: The Sole Catabolic Enzyme Acting on Free D-Aspartate in Mammals. Chem. Biodivers..

[8] Katane M., Kawata T., Nakayama K., Saitoh Y., Kaneko Y., Matsuda S., Saitoh Y., Miyamoto T., Sekine M., Homma H. (2015). Characterization of the Enzymatic and Structural Properties of Human D-Aspartate Oxidase and Comparison with Those of the Rat and Mouse Enzymes. Biol. Pharm. Bull..

[9] Takahashi S. (2020). d-Aspartate oxidase: Distribution, functions, properties, and biotechnological applications. Appl. Microbiol. Biotechnol..

[10] Hashimoto A., Kumashiro S., Nishikawa T., Oka T., Takahashi K., Mito T., Takashima S., Doi N., Mizutani Y., Yamazaki T. (1993). Embryonic Development and Postnatal Changes in Free d-Aspartate and d-Serine in the Human Prefrontal Cortex. J. Neurochem..

[11] Sakai K., Homma H., Lee J.-A., Fukushima T., Santa T., Tashiro K., Iwatsubo T., Imai K. (1998). Emergence of d-aspartic acid in the differentiating neurons of the rat central nervous system. Brain Res..

[12] Hashimoto A., Oka T. (1997). Free d-aspartate and d-serine in the mammalian brain and periphery. Prog. Neurobiol..

[13] Nuzzo T., Sacchi S., Errico F., Keller S., Palumbo O., Florio E., Punzo D., Napolitano F., Copetti M., Carella M. (2017). Decreased free d-aspartate levels are linked to enhanced d-aspartate oxidase activity in the dorsolateral prefrontal cortex of schizophrenia patients. npj Schizophr..

[14] Nuzzo T., Sekine M., Punzo D., Miroballo M., Katane M., Saitoh Y., Galbusera A., Pasqualetti M., Errico F., Gozzi A. (2020). Dysfunctional d-aspartate metabolism in BTBR mouse model of idiopathic autism. Biochim. Biophys. Acta BBA Proteins Proteom..

[15] Errico F., Napolitano F., Squillace M., Vitucci D., Blasi G., De Bartolomeis A., Bertolino A., D’Aniello A., Usiello A. (2013). Decreased levels of d-aspartate and NMDA in the prefrontal cortex and striatum of patients with schizophrenia. J. Psychiatr. Res..

[16] Di Fiore M.M., Santillo A., Falvo S., Longobardi S., Chieffi Baccari G. (2016). Molecular Mechanisms Elicited by d-Aspartate in Leydig Cells and Spermatogonia. Int. J. Mol. Sci..

[17] Di Fiore M.M., Boni R., Santillo A., Falvo S., Gallo A., Esposito S., Chieffi Baccari G. (2019). D-Aspartic Acid in Vertebrate Reproduction: Animal Models and Experimental Designs. Biomolecules.

[18] Raspa M., Mahabir E., Paoletti R., Protti M., Mercolini L., Schiller P., Scavizzi F. (2018). Effects of oral d-aspartate on sperm quality in B6N mice. Theriogenology.

[19] Raspa M., Paoletti R., Mahabir E., Scavizzi F. (2020). d-aspartate treatment in vitro improves mouse sperm fertility in young B6N mice. Theriogenology.

[20] Dunlop D.M., Neidle A., McHale D.M., Lajtha A. (1986). The presence of free D-aspartic acid in rodents and man. Biochem. Biophys. Res. Commun..

[21] Hashimoto A., Oka T., Nishikawa T. (1995). Anatomical Distribution and Postnatal Changes in Endogenous Free D-Aspartate and D-Serine in Rat Brain and Periphery. Eur. J. Neurosci..

[22] De Rosa A., Mastrostefano F., Di Maio A., Nuzzo T., Saitoh Y., Katane M., Isidori A.M., Caputo V., Marotta P., Falco G. (2020). Prenatal expression of d-aspartate oxidase causes early cerebral d-aspartate depletion and influences brain morphology and cognitive functions at adulthood. Amino Acids.

[23] Errico F., Nuzzo T., Carella M., Bertolino A., Usiello A. (2018). The Emerging Role of Altered d-Aspartate Metabolism in Schizophrenia: New Insights from Preclinical Models and Human Studies. Front. Psychiatry.

[24] Punzo D., Errico F., Cristino L., Sacchi S., Keller S., Belardo C., Luongo L., Nuzzo T., Imperatore R., Florio E. (2016). Age-Related Changes in D-Aspartate Oxidase Promoter Methylation Control Extracellular D-Aspartate Levels and Prevent Precocious Cell Death during Brain Aging. J. Neurosci..

[25] Errico F., Cuomo M., Canu N., Caputo V., Usiello A. (2020). New insights on the influence of free d-aspartate metabolism in the mammalian brain during prenatal and postnatal life. Biochim. Biophys. Acta BBA Proteins Proteom..

[26] Errico F., Napolitano F., Nisticò R., Usiello A. (2012). New insights on the role of free d-aspartate in the mammalian brain. Amino Acids.

[27] Ota N., Shi T., Sweedler J.V. (2012). d-Aspartate acts as a signaling molecule in nervous and neuroendocrine systems. Amino Acids.

[28] Wolosker H., D’Aniello A., Snyder S.H. (2000). d-Aspartate disposition in neuronal and endocrine tissues: Ontogeny, biosynthesis and release. Neuroscience.

[29] Schell M.J., Cooper O., Snyder S.H. (1997). D-aspartate localizations imply neuronal and neuroendocrine roles. Proc. Natl. Acad. Sci. USA.

[30] Cuomo M., Keller S., Punzo D., Nuzzo T., Affinito O., Coretti L., Carella M., De Rosa V., Florio E., Boscia F. (2019). Selective demethylation of two CpG sites causes postnatal activation of the Dao gene and consequent removal of d-serine within the mouse cerebellum. Clin. Epigenetics.

[31] Long Z., Homma H., Lee J.-A., Fukushima T., Santa T., Iwatsubo T., Yamada R.-H., Imai K. (1998). Biosynthesis of D-aspartate in mammalian cells. FEBS Lett..

[32] Long Z., Lee J.A., Okamoto T., Nimura N., Imai K., Homma H. (2000). d-Aspartate in a prolactin-secreting clonal strain of rat pituitary tumor cells (GH(3)). Biochem. Biophys. Res. Commun..

[33] Matsuda S., Katane M., Maeda K., Kaneko Y., Saitoh Y., Miyamoto T., Sekine M., Homma H. (2015). Biosynthesis of d-aspartate in mammals: The rat and human homologs of mouse aspartate racemase are not responsible for the biosynthesis of d-aspartate. Amino Acids.

[34] Kim P.M., Duan X., Huang A.S., Liu C.Y., Ming G.-L., Song H., Snyder S.H. (2010). Aspartate racemase, generating neuronal D-aspartate, regulates adult neurogenesis. Proc. Natl. Acad. Sci. USA.

[35] Tanaka-Hayashi A., Hayashi S., Inoue R., Ito T., Konno K., Yoshida T., Watanabe M., Yoshimura T., Mori H. (2015). Is d-aspartate produced by glutamic-oxaloacetic transaminase-1 like 1 (Got1l1): A putative aspartate racemase?. Amino Acids.

[36] Horio M., Ishima T., Fujita Y., Inoue R., Mori H., Hashimoto K. (2013). Decreased levels of free d-aspartic acid in the forebrain of serine racemase (Srr) knock-out mice. Neurochem. Int..

[37] Ito T., Hayashida M., Kobayashi S., Muto N., Hayashi A., Yoshimura T., Mori H. (2016). Serine racemase is involved in d-aspartate biosynthesis. J. Biochem..

[38] Katane M., Kuwabara H., Nakayama K., Saitoh Y., Miyamoto T., Sekine M., Homma H. (2020). Biochemical characterization of d-aspartate oxidase from Caenorhabditis elegans: Its potential use in the determination of free d-glutamate in biological samples. Biochim. Biophys. Acta BBA Proteins Proteom..

[39] Molla G., Chaves-Sanjuan A., Savinelli A., Nardini M., Pollegioni L., Chaves-Sanjuan A. (2019). Structure and kinetic properties of human d -aspartate oxidase, the enzyme-controlling d -aspartate levels in brain. FASEB J..

[40] Puggioni V., Savinelli A., Miceli M., Molla G., Pollegioni L., Sacchi S. (2020). Biochemical characterization of mouse d-aspartate oxidase. Biochim. Biophys. Acta BBA Proteins Proteom..

[41] Katane M., Saitoh Y., Hanai T., Sekine M., Furuchi T., Koyama N., Nakagome I., Tomoda H., Hirono S., Homma H. (2010). Thiolactomycin inhibits d-aspartate oxidase: A novel approach to probing the active site environment. Biochimie.

[42] Van Veldhoven P.P., Brees C., Mannaerts G.P. (1991). d-Aspartate oxidase, a peroxisomal enzyme in liver of rat and man. Biochim. Biophys. Acta BBA Gen. Subj..

[43] Zaar K., Köst H.-P., Schad A., Völkl A., Baumgart E., Fahimi H.D., Baumgart-Vogt E. (2002). Cellular and subcellular distribution of D-aspartate oxidase in human and rat brain. J. Comp. Neurol..

[44] Errico F., Pirro M.T., Affuso A., Spinelli P., De Felice M., D’Aniello A., Di Lauro R. (2006). A physiological mechanism to regulate d-aspartic acid and NMDA levels in mammals revealed by D-aspartate oxidase deficient mice. Gene.

[45] Sacchi S., Bernasconi M., Martineau M., Mothet J.-P., Ruzzene M., Pilone M.S., Pollegioni L., Molla G. (2008). pLG72 Modulates Intracellular D-Serine Levels through Its Interaction with D-Amino Acid Oxidase: Effect on schizophrenia susceptibility. J. Biol. Chem..

[46] Keller S., Punzo D., Cuomo M., Affinito O., Coretti L., Sacchi S., Florio E., Lembo F., Carella M., Copetti M. (2018). DNA methylation landscape of the genes regulating D-serine and D-aspartate metabolism in post-mortem brain from controls and subjects with schizophrenia. Sci. Rep..

[47] Florio E., Keller S., Coretti L., Affinito O., Scala G., Errico F., Fico A., Boscia F., Sisalli M.J., Reccia M.G. (2017). Tracking the evolution of epialleles during neural differentiation and brain development: D-Aspartate oxidase as a model gene. Epigenetics.

[48] Jagannath V., Marinova Z., Monoranu C.-M., Walitza S., Grünblatt E. (2017). Expression of D-Amino Acid Oxidase (DAO/DAAO) and D-Amino Acid Oxidase Activator (DAOA/G72) during Development and Aging in the Human Post-mortem Brain. Front. Neuroanat..

[49] Fagg G.E., Matus A. (1984). Selective association of N-methyl aspartate and quisqualate types of L-glutamate receptor with brain postsynaptic densities. Proc. Natl. Acad. Sci. USA.

[50] Ransom R.W., Stec N.L. (1988). Cooperative Modulation of [3H]MK-801 Binding to the N-Methyl-d-Aspartate Receptor-Ion Channel Complex by l-Glutamate, Glycine, and Polyamines. J. Neurochem..

[51] Errico F., Nisticò R., Palma G., Federici M., Affuso A., Brilli E., Topo E., Centonze D., Bernardi G., Bozzi Y. (2008). Increased levels of d-aspartate in the hippocampus enhance LTP but do not facilitate cognitive flexibility. Mol. Cell. Neurosci..

[52] Krashia P., LeDonne A., Nobili A., Cordella A., Errico F., Usiello A., D’Amelio M., Mercuri N.B., Guatteo E., Carunchio I. (2016). Persistent elevation of D-Aspartate enhances NMDA receptor-mediated responses in mouse substantia nigra pars compacta dopamine neurons. Neuropharmacology.

[53] Errico F., Nisticò R., Napolitano F., Oliva A.B., Romano R., Barbieri F., Florio T., Russo C., Mercuri N.B., Usiello A. (2011). Persistent increase of d-aspartate in d-aspartate oxidase mutant mice induces a precocious hippocampal age-dependent synaptic plasticity and spatial memory decay. Neurobiol. Aging.

[54] Gong X.-Q., Frandsen A., Lu W.-Y., Wan Y., Zabek R.L., Pickering D.S., Bai D. (2005). D -Aspartate and NMDA, but not L -aspartate, block AMPA receptors in rat hippocampal neurons. Br. J. Pharmacol..

[55] Molinaro G., Pietracupa S., Di Menna L., Pescatori L., Usiello A., Battaglia G., Nicoletti F., Bruno V.M.G. (2010). d-Aspartate activates mGlu receptors coupled to polyphosphoinositide hydrolysis in neonate rat brain slices. Neurosci. Lett..

[56] Sacchi S., De Novellis V., Paolone G., Nuzzo T., Iannotta M., Belardo C., Squillace M., Bolognesi P., Rosini E., Motta Z. (2017). Olanzapine, but not clozapine, increases glutamate release in the prefrontal cortex of freely moving mice by inhibiting D-aspartate oxidase activity. Sci. Rep..

[57] Boccella S., Vacca V., Errico F., Marinelli S., Squillace M., Guida F., Di Maio A., Vitucci D., Palazzo E., De Novellis V. (2015). D-Aspartate Modulates Nociceptive-Specific Neuron Activity and Pain Threshold in Inflammatory and Neuropathic Pain Condition in Mice. BioMed Res. Int..

[58] D’Aniello S., Somorjai I.M.L., Garcia-Fernàndez J., Topo E., D’Aniello A. (2010). D-Aspartic acid is a novel endogenous neurotransmitter. FASEB J..

[59] Anderson C.M., Bridges R.J., Chamberlin A.R., Shimamoto K., Yasuda-Kamatani Y., Swanson R.A. (2001). Differing effects of substrate and non-substrate transport inhibitors on glutamate uptake reversal. J. Neurochem..

[60] Bak L.K., Schousboe A., Waagepetersen H.S. (2003). Characterization of depolarization-coupled release of glutamate from cultured mouse cerebellar granule cells using dl-threo-β-benzyloxyaspartate (DL-TBOA) to distinguish between the vesicular and cytoplasmic pools. Neurochem. Int..

[61] Guida F., Luongo L., Marmo F., Romano R., Iannotta M., Napolitano F., Belardo C., Marabese I., Aniello A.D., De Gregorio D. (2015). Palmitoylethanolamide reduces pain-related behaviors and restores glutamatergic synapses homeostasis in the medial prefrontal cortex of neuropathic mice. Mol. Brain.

[62] Palacín M., Estévez R., Bertran J., Zorzano A. (1998). Molecular Biology of Mammalian Plasma Membrane Amino Acid Transporters. Physiol. Rev..

[63] Arkhipova V., Trinco G., Ettema T.W., Jensen S., Slotboom D.J., Guskov A. (2019). Binding and transport of D-aspartate by the glutamate transporter homolog GltTk. eLife.

[64] Huang A.S., Beigneux A., Weil Z.M., Kim P.M., Molliver M.E., Blackshaw S., Nelson R.J., Young S.G., Snyder S.H. (2006). D-Aspartate Regulates Melanocortin Formation and Function: Behavioral Alterations in D-Aspartate Oxidase-Deficient Mice. J. Neurosci..

[65] Weil Z.M., Huang A.S., Beigneux A., Kim P.M., Molliver M.E., Blackshaw S., Young S.G., Nelson R.J., Snyder S.H. (2006). Behavioural alterations in male mice lacking the gene for d-aspartate oxidase. Behav. Brain Res..

[66] Jami S.A., Cameron S., Wong J.M., Daly E.R., McAllister A.K., Gray J.A. (2020). Increased excitation-inhibition balance due to a loss of GABAergic synapses in the serine racemase knockout model of NMDA receptor hypofunction. bioRxiv.

[67] Errico F., Bonito-Oliva A., Bagetta V., Vitucci D., Romano R., Zianni E., Napolitano F., Marinucci S., Di Luca M., Calabresi P. (2011). Higher free D-aspartate and N-methyl-d-aspartate levels prevent striatal depotentiation and anticipate l-DOPA-induced dyskinesia. Exp. Neurol..

[68] Errico F., Rossi S., Napolitano F., Catuogno V., Topo E., Fisone G., D’Aniello A., Centonze D., Usiello A. (2008). D-Aspartate Prevents Corticostriatal Long-Term Depression and Attenuates Schizophrenia-Like Symptoms Induced by Amphetamine and MK-801. J. Neurosci..

[69] Kitamura A., Hojo Y., Ikeda M., Karakawa S., Kuwahara T., Kim J., Soma M., Kawato S., Tsurugizawa T. (2018). Ingested d-Aspartate Facilitates the Functional Connectivity and Modifies Dendritic Spine Morphology in Rat Hippocampus. Cereb. Cortex.

[70] Bauer D., Hamacher K., Bröer S., Pauleit D., Palm C., Zilles K., Coenen H.H., Langen K.-J. (2005). Preferred stereoselective brain uptake of d-serine — a modulator of glutamatergic neurotransmission. Nucl. Med. Biol..

[71] Langen K.-J., Hamacher K., Bauer D., Bröer S., Pauleit D., Herzog H., Floeth F., Zilles K., Coenen H.H. (2005). Preferred Stereoselective Transport of the D-isomer of cis-4-[18F]fluoro-proline at the Blood–Brain Barrier. Br. J. Pharmacol..

[72] Errico F., Nistico R., Di Giorgio A., Squillace M., Vitucci D., Galbusera A., Piccinin S., Mango D., Fazio L., Middei S. (2014). Free D-aspartate regulates neuronal dendritic morphology, synaptic plasticity, gray matter volume and brain activity in mammals. Transl. Psychiatry.

[73] Topo E., Soricelli A., Di Maio A., D’Aniello E., Di Fiore M.M., D’Aniello A. (2009). Evidence for the involvement of d-aspartic acid in learning and memory of rat. Amino Acids.

[74] Luo T., Wu W.-H., Chen B.-S. (2011). NMDA receptor signaling: Death or survival?. Front. Biol..

[75] Hardingham G.E., Bading H. (2003). The Yin and Yang of NMDA receptor signalling. Trends Neurosci..

[76] Nuzzo T., Feligioni M., Cristino L., Pagano I., Marcelli S., Iannuzzi F., Imperatore R., D’Angelo L., Petrella C., Carella M. (2019). Free D-aspartate triggers NMDA receptor-dependent cell death in primary cortical neurons and perturbs JNK activation, Tau phosphorylation, and protein SUMOylation in the cerebral cortex of mice lacking d-aspartate oxidase activity. Exp. Neurol..

[77] Di Giorgi-Gerevini V., Melchiorri D., Battaglia G., Ricci-Vitiani L., Ciceroni C., Busceti C.L., Biagioni F., Iacovelli L., Canudas A.M., Parati E. (2005). Endogenous activation of metabotropic glutamate receptors supports the proliferation and survival of neural progenitor cells. Cell Death Differ..

[78] Ikonomidou C. (2009). Triggers of apoptosis in the immature brain. Brain Dev..

[79] Jansson L.C., Åkerman K.E. (2014). The role of glutamate and its receptors in the proliferation, migration, differentiation and survival of neural progenitor cells. J. Neural Transm..

[80] Komuro H., Rakic P. (1993). Modulation of neuronal migration by NMDA receptors. Science.

[81] Coyle J.T. (2012). NMDA Receptor and Schizophrenia: A Brief History. Schizophr. Bull..

[82] Gonzalez J., Jurado-Coronel J.C., Ávila M.F., Sabogal A., Capani F., Barreto G.E. (2014). NMDARs in neurological diseases: A potential therapeutic target. Int. J. Neurosci..

[83] Krivoy A., Fischel T., Weizman A. (2008). The possible involvement of metabotropic glutamate receptors in schizophrenia. Eur. Neuropsychopharmacol..

[84] Matosin N., Fernandez F.M., Lum J.S., Newell K.A. (2017). Shifting towards a model of mGluR5 dysregulation in schizophrenia: Consequences for future schizophrenia treatment. Neuropharmacology.

[85] Moghaddam B., Javitt D.C. (2012). From Revolution to Evolution: The Glutamate Hypothesis of Schizophrenia and its Implication for Treatment. Neuropsychopharmacology.

[86] Ribeiro F.M., Paquet M., Cregan S.P., Ferguson S.S.G. (2010). Group I Metabotropic Glutamate Receptor Signalling and its Implication in Neurological Disease. CNS Neurol. Disord. Drug Targets.

[87] Gardoni F., Bellone C. (2015). Modulation of the glutamatergic transmission by Dopamine: A focus on Parkinson, Huntington and Addiction diseases. Front. Cell. Neurosci..

[88] Mellone M., Gardoni F. (2018). Glutamatergic mechanisms in l-DOPA-induced dyskinesia and therapeutic implications. J. Neural Transm..

[89] Nuzzo T., Punzo D., Devoto P., Rosini E., Paciotti S., Sacchi S., Li Q., Thiolat M.-L., Véga C., Carella M. (2019). The levels of the NMDA receptor co-agonist D-serine are reduced in the substantia nigra of MPTP-lesioned macaques and in the cerebrospinal fluid of Parkinson’s disease patients. Sci. Rep..

[90] Nuzzo T., Miroballo M., Casamassa A., Mancini A., Gaetani L., Nistico R., Eusebi P., Katane M., Homma H., Calabresi P. (2020). Cerebrospinal fluid and serum d-serine concentrations are unaltered across the whole clinical spectrum of Alzheimer’s disease. Biochim. Biophys. Acta Proteins. Proteom..

[91] Li Y., Han H., Yin J., Li T., Yin Y. (2018). Role of D-aspartate on biosynthesis, racemization, and potential functions: A mini-review. Anim. Nutr..

[92] Yamamoto A., Tanaka H., Ishida T., Horiike K. (2010). d-Aspartate Oxidase Localisation in Pituitary and Pineal Glands of the Female Pig. J. Neuroendocr..

[93] Topo E., Soricelli A., D’Aniello A., Ronsini S., D’Aniello A. (2009). The role and molecular mechanism of D-aspartic acid in the release and synthesis of LH and testosterone in humans and rats. Reprod. Biol. Endocrinol..

[94] Burrone L., Di Giovanni M., Di Fiore M.M., Chieffi Baccari G., Santillo A. (2010). Effects of D-Aspartate Treatment on D-Aspartate Oxidase, Superoxide Dismutase, and Caspase 3 Activities in Frog (*Rana esculenta*) Tissues. Chem. Biodivers..

[95] Di Giovanni M., Burrone L., Chieffi Baccari G., Topo E., Santillo A. (2010). Distribution of free D-aspartic acid and D-aspartate oxidase in frogRana esculentatissues. J. Exp. Zool. Part A Ecol. Genet. Physiol..

[96] Han H., Miyoshi Y., Koga R., Mita M., Konno R., Hamase K. (2015). Changes in d-aspartic acid and d-glutamic acid levels in the tissues and physiological fluids of mice with various d-aspartate oxidase activities. J. Pharm. Biomed. Anal..

[97] Hamase K., Konno R., Morikawa A., Zaitsu K. (2005). Sensitive Determination of D-Amino Acids in Mammals and the Effect of D-Amino-Acid Oxidase Activity on Their Amounts. Biol. Pharm. Bull..

[98] Han H., Miyoshi Y., Ueno K., Okamura C., Tojo Y., Mita M., Lindner W., Zaitsu K., Hamase K. (2011). Simultaneous determination of d-aspartic acid and d-glutamic acid in rat tissues and physiological fluids using a multi-loop two-dimensional HPLC procedure. J. Chromatogr. B.

[99] Morikawa A., Hamase K., Inoue T., Konno R., Niwa A., Zaitsu K. (2001). Determination of free D-aspartic acid, D-serine and D-alanine in the brain of mutant mice lacking D-amino acid oxidase activity. J. Chromatogr. B, Biomed. Sci. Appl..

[100] Miyoshi Y., Koga R., Oyama T., Han H., Ueno K., Masuyama K., Itoh Y., Hamase K. (2012). HPLC analysis of naturally occurring free d-amino acids in mammals. J. Pharm. Biomed. Anal..

[101] Imai K., Fukushima T., Hagiwara K., Santa T. (1995). Occurrence ofD-aspartic acid in rat brain pineal gland. Biomed. Chromatogr..

[102] Sakai K., Homma H., Lee J.-A., Fukushima T., Santa T., Tashiro K., Iwatsubo T., Imai K. (1997). D-Aspartic Acid Localization during Postnatal Development of Rat Adrenal Gland. Biochem. Biophys. Res. Commun..

[103] D·Aniello A., Di Fiore M.M., Fisher G.H., Milone A., Seleni A., D’Aniello S., Perna A.F., Ingrosso D. (2000). Occurrence of D-aspartic acid and N-methyl-D-aspartic acid in rat neuroendocrine tissues and their role in the modulation of luteinizing hormone and growth hormone release. FASEB J..

[104] Wang H., Wolosker H., Pevsner J., Snyder S.H., Selkoe D.J. (2000). Regulation of rat magnocellular neurosecretory system by D-aspartate: Evidence for biological role(s) of a naturally occurring free D-amino acid in mammals. J. Endocrinol..

[105] Pampillo M., Scimonelli T., Bottino M.C., Duvilanski B.H., McCann S.M., Seilicovich A., Lasaga M. (2002). The effect of D-aspartate on luteinizing hormone-releasing hormone, α-melanocyte-stimulating hormone, GABA and dopamine release. NeuroReport.

[106] D’Aniello A. (2007). d-Aspartic acid: An endogenous amino acid with an important neuroendocrine role. Brain Res. Rev..

[107] Hamase K., Homma H., Takigawa Y., Fukushima T., Santa T., Imai K. (1997). Regional distribution and postnatal changes of d-amino acids in rat brain. Biochim. Biophys. Acta BBA Gen. Subj..

[108] Han H., Miyoshi Y., Oyama T., Konishi R., Mita M., Hamase K. (2011). Enantioselective micro-2D-HPLC determination of aspartic acid in the pineal glands of rodents with various melatonin contents. J. Sep. Sci..

[109] Ishio S., Yamada H., Hayashi M., Yatsushiro S., Noumi T., Yamaguchi A., Moriyama Y. (1998). d-Aspartate modulates melatonin synthesis in rat pinealocytes. Neurosci. Lett..

[110] Karakawa S., Shimbo K., Yamada N., Mizukoshi T., Miyano H., Mita M., Lindner W., Hamase K. (2015). Simultaneous analysis of d-alanine, d-aspartic acid, and d-serine using chiral high-performance liquid chromatography-tandem mass spectrometry and its application to the rat plasma and tissues. J. Pharm. Biomed. Anal..

[111] Tsunoda M., Kato M., Fukushima T., Santa T., Homma H., Yanai H., Soga T., Imai K. (1999). Determination of aspartic acid enantiomers in bio-samples by capillary electrophoresis. Biomed. Chromatogr..

[112] Yatsushiro S., Yamada H., Kozaki S., Kumon H., Michibata H., Yamamoto A., Moriyama Y. (1997). L-aspartate but not the D form is secreted through microvesicle-mediated exocytosis and is sequestered through Na+-dependent transporter in rat pinealocytes. J. Neurochem..

[113] Takigawa Y., Homma H., Lee J.-A., Fukushima T., Santa T., Iwatsubo T., Imai K. (1998). d-Aspartate Uptake into Cultured Rat Pinealocytes and the Concomitant Effect onl-Aspartate Levels and Melatonin Secretion. Biochem. Biophys. Res. Commun..

[114] Lee J.-A., Long Z., Nimura N., Iwatsubo T., Imai K., Homma H. (2001). Localization, Transport, and Uptake of -Aspartate in the Rat Adrenal and Pituitary Glands. Arch. Biochem. Biophys..

[115] Long Z., Sekine M., Adachi M., Furuchi T., Imai K., Nimura N., Homma H. (2002). Cell density inversely regulates d- and l-aspartate levels in rat pheochromocytoma MPT1 cells. Arch. Biochem. Biophys..

[116] Nakatsuka S., Hayashi M., Muroyama A., Otsuka M., Kozaki S., Yamada H., Moriyama Y. (2001). d-Aspartate Is Stored in Secretory Granules and Released through a Ca^2+^-dependent Pathway in a Subset of Rat Pheochromocytoma PC12 Cells. J. Biol. Chem..

[117] Santillo A., Falvo S., Chieffi P., Burrone L., Chieffi Baccari G., Longobardi S., Di Fiore M.M. (2014). d-aspartate affects NMDA receptor-extracellular signal–regulated kinase pathway and upregulates androgen receptor expression in the rat testis. Theriogenology.

[118] Yamada H., Yatsushiro S., Yamamoto A., Hayashi M., Nishi T., Futai M., Yamaguchi A., Moriyama Y. (1997). Functional expression of a GLT-1 type Na+-dependent glutamate transporter in rat pinealocytes. J. Neurochem..

[119] Hamase K., Morikawa A., Zaitsu K. (2002). d-Amino acids in mammals and their diagnostic value. J. Chromatogr. B.

[120] Topo E., Fisher G., Sorricelli A., Errico F., Usiello A., D’Aniello A. (2010). Thyroid Hormones and D-Aspartic Acid, D-Aspartate Oxidase, D-Aspartate Racemase, H2O2, and ROS in Rats and Mice. Chem. Biodivers..

[121] Lee J.-A., Homma H., Tashiro K., Iwatsubo T., Imai K. (1999). d-Aspartate localization in the rat pituitary gland and retina. Brain Res..

[122] Boni R., Santillo R., Macchia G., Spinelli P., Ferrandino G., D’Aniello A. (2006). d-Aspartate and reproductive activity in sheep. Theriogenology.

[123] D’Aniello G., Tolino A., D’Aniello A., Errico F., Fisher G.H., Di Fiore M.M. (2000). The role of D-aspartic acid and N-methyl-D-aspartic acid in the regulation of prolactin release. Endocrinology.

[124] Raucci F., D’Aniello A., Di Fiore M.M. (2014). Stimulation of androgen production by D-aspartate through the enhancement of StAR, P450scc and 3beta-HSD mRNA levels in vivo rat testis and in culture of immature rat Leydig cells. Steroids.

[125] Burrone L., Santillo A., Pinelli C., Chieffi Baccari G., Di Fiore M.M. (2012). Induced synthesis of P450 aromatase and 17beta-estradiol by D-aspartate in frog brain. J. Exp. Biol..

[126] Di Fiore M.M., Santillo A., Falvo S., Chieffi Baccari G., Venditti M., Russo F.D.G., Lispi M., D’Aniello A. (2018). Sex hormone levels in the brain of d -aspartate-treated rats. Comptes Rendus Biol..

[127] Santillo A., Pinelli C., Burrone L., Chieffi Baccari G., Di Fiore M.M. (2013). d-Aspartic acid implication in the modulation of frog brain sex steroid levels. Gen. Comp. Endocrinol..

[128] Falvo S., Di Fiore M.M., Burrone L., Chieffi Baccari G., Longobardi S., Santillo A. (2016). Androgen and oestrogen modulation by D-aspartate in rat epididymis. Reprod. Fertil. Dev..

[129] Nagata Y., Homma H., Matsumoto M., Imai K. (1999). Stimulation of steroidogenic acute regulatory protein (STAR) gene expression by D-aspartate in rat Leydig cells. FEBS Lett..

[130] Di Nisio A., De Toni L., Ferigo M., Rocca M.S., Speltra E., Ferlin A., Foresta C. (2015). d-Aspartic acid stimulates steroidogenesis through the delay of LH receptor internalization in a mammalian Leydig cell line. J. Endocrinol. Investig..

[131] Bhat G.K., Mahesh V.B., Chu Z.W., Chorich L.P., Zamorano P.L., Brann D.W. (1995). Localization of the N-Methyl-D-Aspartate R1 Receptor Subunit in Specific Anterior Pituitary Hormone Cell Types of the Female Rat. Neuroendocrinology.

[132] Storto M., Sallese M., Salvatore L., Poulet R., Condorelli D.F., Dell’Albani P., Marcello M.F., Romeo R., Piomboni P., Barone N. (2001). Expression of metabotropic glutamate receptors in the rat and human testis. J. Endocrinol..

[133] Di Giovanni M., Topo E., Santillo A., D’Aniello A., Chieffi Baccari G. (2009). d-Aspartate binding sites in rat Harderian gland. Amino Acids.

[134] Santillo A., Falvo S., Chieffi P., Di Fiore M.M., Senese R., Chieffi Baccari G. (2015). D-Aspartate Induces Proliferative Pathways in Spermatogonial GC-1 Cells. J. Cell. Physiol..

[135] Santillo A., Falvo S., Di Fiore M.M., Russo F.D.G., Chieffi P., Usiello A., Pinelli C., Chieffi Baccari G. (2019). AMPA receptor expression in mouse testis and spermatogonial GC-1 cells: A study on its regulation by excitatory amino acids. J. Cell. Biochem..

[136] Tomita K., Tanaka H., Kageyama S., Nagasawa M., Wada A., Murai R., Kobayashi K., Hanada E., Agata Y., Kawauchi A. (2016). The Effect of d-Aspartate on Spermatogenesis in Mouse Testis1. Biol. Reprod..

[137] Santillo A., Venditti M., Minucci S., Chieffi Baccari G., Falvo S., Rosati L., Di Fiore M.M. (2019). D-Asp upregulates PREP and GluA2/3 expressions and induces p-ERK1/2 and p-Akt in rat testis. Reproduction.

[138] Venditti M., Santillo A., Falvo S., Di Fiore M.M., Chieffi Baccari G., Minucci S. (2020). D-Aspartate Upregulates DAAM1 Protein Levels in the Rat Testis and Induces Its Localization in Spermatogonia Nucleus. Biomolecules.

[139] Boni R., Gallo A., Cecchini S. (2016). Kinetic activity, membrane mitochondrial potential, lipid peroxidation, intracellular pH and calcium of frozen/thawed bovine spermatozoa treated with metabolic enhancers. Andrology.

[140] D’Aniello G., Ronsini S., Guida F., Spinelli P., D’Aniello A. (2005). Occurrence of D-aspartic acid in human seminal plasma and spermatozoa: Possible role in reproduction. Fertil. Steril..

[141] D’Aniello A., Ronsini S., Notari T., Grieco N., Infante V., D’Angel N., Mascia F., Di Fiore M.M., Fisher G., D’Aniello A. (2012). D-Aspartate, a Key Element for the Improvement of Sperm Quality. Adv. Sex. Med..

[142] Giacone F., Condorelli R.A., Mongioì L.M., Bullara V., La Vignera S., Calogero A.E. (2016). In vitro effects of zinc, D-aspartic acid, and coenzyme-Q10 on sperm function. Endocrine.

[143] Fink J., Schoenfeld B.J., Nakazato K. (2018). The role of hormones in muscle hypertrophy. Physician Sportsmed..

[144] Willoughby D.S., Leutholtz B. (2013). d-Aspartic acid supplementation combined with 28 days of heavy resistance training has no effect on body composition, muscle strength, and serum hormones associated with the hypothalamo-pituitary-gonadal axis in resistance-trained men. Nutr. Res..

[145] Melville G.W., Siegler J.C., Marshall P.W. (2015). Three and six grams supplementation of d-aspartic acid in resistance trained men. J. Int. Soc. Sports Nutr..

[146] Melville G., Siegler J.C., Marshall P.W.M. (2017). The effects of d-aspartic acid supplementation in resistance-trained men over a three month training period: A randomised controlled trial. PLoS ONE.

[147] De Rosa V., Secondo A., Pannaccione A., Ciccone R., Formisano L., Guida N., Crispino R., Fico A., Polishchuk R., D’Aniello A. (2018). D-Aspartate treatment attenuates myelin damage and stimulates myelin repair. EMBO Mol. Med..

[148] Nicoletti C.G., Monteleone F., Marfia G.A., Usiello A., Buttari F., Centonze D., Mori F. (2019). Oral D-Aspartate enhances synaptic plasticity reserve in progressive multiple sclerosis. Mult. Scler. J..

